# Language statistics as a window into mental representations

**DOI:** 10.1038/s41598-022-12027-5

**Published:** 2022-05-16

**Authors:** Fritz Günther, Luca Rinaldi

**Affiliations:** 1 Department of Psychology, Humboldt-Universität zu Berlin, Berlin, Germany; 2 Department of Brain and Behavioral Sciences, University of Pavia, Pavia, Italy; 3 Cognitive Psychology Unit, IRCCS Mondino Foundation, Pavia, Italy

**Keywords:** Psychology, Human behaviour

## Abstract

Large-scale linguistic data is nowadays available in abundance. Using this source of data, previous research has identified redundancies between the statistical structure of natural language and properties of the (physical) world we live in. For example, it has been shown that we can gauge city sizes by analyzing their respective word frequencies in corpora. However, since natural language is always produced by human speakers, we point out that such redundancies can only come about indirectly and should necessarily be restricted cases where human representations largely retain characteristics of the physical world. To demonstrate this, we examine the statistical occurrence of words referring to body parts in very different languages, covering nearly 4 billions of native speakers. This is because the convergence between language and physical properties of the stimuli clearly breaks down for the human body (i.e., more relevant and functional body parts are not necessarily larger in size). Our findings indicate that the human body as extracted from language does not retain its actual physical proportions; instead, it resembles the distorted human-like figure known as the sensory homunculus, whose form depicts the amount of cortical area dedicated to sensorimotor functions of each body part (and, thus, their relative functional relevance). This demonstrates that the surface-level statistical structure of language opens a window into how humans represent the world they live in, rather than into the world itself.

## Introduction

Imagine a group of alien scientists in the distant future, specializing in research on humankind. Their only source material on the human species is a vast collection of text contained in digital documents, which they recovered from a capsule sent into space a long time ago. Other than that, they have no archeological sites, image material, or any other type of information. They have the ability to decode human language (that is, they know about individual word meanings, and could in principle read the entire text), but the sheer amount of data makes it virtually impossible to read the entire material and meticulously analyze its content. (Without the ability to also actually understand language, it remains doubtful whether a statistical analysis of text alone allows our hypothetical scientists to identify the meanings and referents of the symbols presented to them^1^). Therefore, to rapidly gain information about humankind and the way they experienced the world they live in, this group of alien scientists begins their endeavor with a statistical analysis of the language material. What can they learn from such an analysis? Despite arising from a thought experiment, the question about the very types of information that can be recovered from language has captivated philosophers, linguists and cognitive scientists for the past century (e.g.^2^; see also^3,4^ for recent overviews). This question has indeed very distant theoretical origins that can be traced back to the symbol grounding problem^5^ and to the subsequent debate in the scientific literature between symbolic and embodied accounts of cognition^6^, igniting fervent discussions in psychology^7^, artificial intelligence^8^ and linguistics^9^.

Previous research has shown that the type of knowledge that can be extracted from natural language data—even from its surface-level statistical structure alone, without semantic analyses of its content—is surprisingly extensive. For example, word frequencies are positively correlated with the population sizes of cities^10^, and statistical analyses of natural language data even reveal the real geographical distances between places^10,11^ or the typical spatial arrangement of objects^12^. Even more striking evidence in this respect comes from congenitally blind individuals who never had any visual experience but can exploit linguistic information, which enables them to linguistically categorize colors and correctly assign colors to objects^13^ and to differentiate different kinds of “seeing”, such as peeking versus staring^14^.

At a first glance, this opens an interesting hypothesis on the type of information encoded in language data: Does natural language allow us to re-construct or at least make informed guesses about some proprieties of the (physical) world surrounding us, since speakers use language to communicate about this world? If natural language data allows us to predict the location of archeological sites^11^, can our hypothetical alien scientist use statistical analyses to paint a picture of how Earth and the beings inhabiting it looked like? What is actually encoded in this data?

While this is an appealing prospect, we argue for caution. Language is after all an artefact produced by humans, and thus inherently subject to human biases and distortions. This is acknowledged in the aforementioned literature, where the findings are explained via redundancies between language and how humans perceive the world^15^. For instance, the relationship between population sizes of cities and word frequency may be triggered by the fact that “cities that are populated more are debated more” (i.e., because they are more salient and relevant than less populated cities^10^). However, despite the acknowledged interpretation that relevance could also be a factor, it has never been empirically tested. In fact, in the presented test cases (such as city sizes, geographical distances, and object colors), the physical properties of entities in the outside world on the one hand and the human representation of these entities on the other hand are typically highly conflated. As demonstrated in the study by Recchia and Louwerse^11^, this in principle still suggests the possibility of an indirect route, where the physical world can be re-constructed via (at least partially) structurally similar human representations of it that end up being encoded in language. The purpose of the present study is to demonstrate that this is not generally the case, but only works in a subset of cases. To this end, it is necessary to investigate a case where human representations of an entity are completely dissociated from the actual physical properties of the same entity.

As a prime example for such a dissociation, we take the human body (and more specifically, the size of individual body parts), arguably one of the most salient stimuli we are exposed to throughout our lifetime. This is because the size of the different body parts is far from proportional to their (functional) relevance. This is prominently testified by Penfield’s cortical homunculus, an emblematic figure that appears in nearly each textbook of biology, physiology, and neuroscience (^16^; see also ^17^). The homunculus depicts a stark contrast between the actual physical size of body parts (a stimulus our visual system is constantly exposed to) and their representational sizes, which are indicative about the density of receptors for those body parts (and, consequently, about the functional relevance of body parts). For instance, in the case of the somatosensory homunculus, the size of the anatomical body part would not be related to the amount of the cortex dedicated to that body part. The amount of the cortex assigned for one body part rather reflects the density of cutaneous tactile receptors: despite lips occupying a small surface area, they have a greater density of receptors compared to other body parts such as shoulders or forearms.

Here, we investigate surface-level language statistics in the form of word frequencies, likely the easiest and most accessible—yet also one of the most revealing—index we can extract from language^18,19^. Frequency is generally taken as a measure for familiarity with a word^20^ and even its referent^21^, and thus of how relevant the word/concept is in our experience. As suggested in the study by Louwerse and Zwaan^10^, this might allow for (indirect) inferences about the physical magnitude of entities, as exemplified by the population sizes of cities. The main objective of the present study is to test whether word frequencies of body parts capture their physical size (the surface area of body parts) or rather their representational size (as indexed by the somatosensory homunculus). However, before turning to this test case, we first ensure the robustness and generalizability of the results by Louwerse and Zwaan^10^ by demonstrating that the possibility to recover physical size from language through an analysis of word frequencies is not restricted to city sizes only.

## Study 1: Word frequencies encode the relevance of geographical entities

While previous studies focused on cities population size only, here we aimed at replicating this relationship and extending it also to other types of physical size, namely countries’ total population and rivers lengths. In all cases, we investigated the English (UK), French, German, and Italian languages, in order to mitigate the influence of idiosyncratic patterns specific to each language. We collected (a) lengths of the ten longest rivers and (b) the names and population sizes of the 40 most populous cities within each of the four countries (United Kingdom, France, Germany, and Italy), as well as (c) the population sizes of all European countries, and then tested whether these proxies for relevance (i.e., more relevant geographical entities will be mentioned more in language) predicted their respective word frequencies.

### Methods

All the data, materials and codes have been archived in the free Open Science Framework https://osf.io/6zk8s/?view_only=1e7eae8fcf534c32bbf796be7e59930f. These files include all materials and specific instructions to retrace and replicate all measures described here and to reproduce the current findings.

For the river study, we collected lengths of the ten longest rivers within each of the four countries (United Kingdom, France, Germany, and Italy) and their names in the respective language (such as *Donau* for *Danube*) from the respective Wikipedia pages (https://de.wikipedia.org/wiki/Liste_von_Flüssen_in_Deutschland; https://it.wikipedia.org/wiki/Fiumi_d’Italia; https://en.wikipedia.org/wiki/Longest_rivers_of_the_United_Kingdom; https://fr.wikipedia.org/wiki/Liste_des_fleuves_de_France). The river Rhine appeared two times, in German and French. For the city study, we collected the names and population sizes of the 40 most populous cities in the UK, France, Italy, and Germany, respectively (all in their native spelling, such as *Firenze* for *Florence*). The data were collected from the Office for National Statistics 2011 Census (UK), Institut National de la Statistique et des Études Économiques 2006 Census (France), Istituto Nazionale di Statistica 2008 Census (Italy), and Statistische Landes- und Bundesämter 2010 Census (Germany). For the country study, we collected the population sizes of all European countries from the CIA 2008 world factbook^22^ (except for Kosovo, which is not listed in the 2008 factbook). Since the distributions of these physical magnitude measures are heavily right-skewed, these predictor values were all log-transformed.

We collected population size data from the 2006 to 2011 timeframe to ensure a better overlap with the corpora from which the word frequencies were derived: The large parsed and lemmatized web-collected WaCKy corpora^23^, which were constructed by crawling .uk, .fr, .it, and .de domains, respectively, using random search terms (see Table 1). Thus, employing the same algorithm for all four languages, these corpora were collected from independent sources generated by different speaker populations. This is especially desirable for the between-item studies, as we expect speakers of a language to talk more about their own than other countries’ cities or rivers. The systematically evaluated WaCKy corpora are currently only available for these four languages (see https://wacky.sslmit.unibo.it).

Word frequencies for the city names were extracted from their corresponding corpora (e.g., itWaC frequencies for the 40 Italian cities). To account for corpus size differences, we computed word frequencies per million words, which were then logarithmized^24^.

**Table 1:** The languages employed and the corpora from which the word frequencies were extracted.

Language family	Sub-family	Language	Corpus size (mio.)	Corpus name
Indo-European	Germanic	**English**	**1909**	**ukWaC**
		**German**	**1339**	**deWaC**
		Dutch	2539	nlTenTen14
	Romance	**French**	**1331**	**frWaC**
		**Italian**	**1556**	**itWaC**
		Spanish	98	SpanishWaC
		Portuguese	3896	ptTenTen11
	Italic	Latin	11	LatinISE
	Slavic	Russian	14,554	ruTenTen11
		Polish	7716	plTenTen12
		Czech	10,502	csTenTen17
		Croatian	1210	hrWaC
	Baltic	Latvian	530	LatvianWaC
	Hellenic	Greek	124	gkWaC
	Indo-Aryan	Urdu	53	UrduWaC
		Hindi	108	HindiWaC
		Bengali	12	bnWaC
Uralic	Finno-Ugric	Hungarian	2573	huTenTen12
Turkic	Oghuz	Turkish	33	trWaC
Afro-Asiatic	Semitic	Arabic	7476	arTenTen12
		Hebrew	48	hebWaC
		Amharic	26	amWaC
	Cushitic	Somali	72	soWaC
Niger-Congo	Bantu	Swahili	18	SwahiliWaC
	Volta-Niger	Yoruba	3	YorubaWaC
Dravidian	Southern	Tamil	27	TamilWaC
Austronesian	Malayo-Polynesian	Malay	183	MalaysianWaC
		Tagalog (Filipino)	198	tlTenTen19
Sino-Tibetan	Sinitic	Chinese (simplified)	13,531	zhTenTen17
Japonic	–	Japanese (Kanji)	337	jpWaC

The languages investigated in Study 1 and 2 are displayed in boldface; all other languages were added in Study 3. Corpus Size refers to the number of tokens in the corpora after non-alphabetic characters and annotation tags have been removed.

### Results

**Figure 1:**
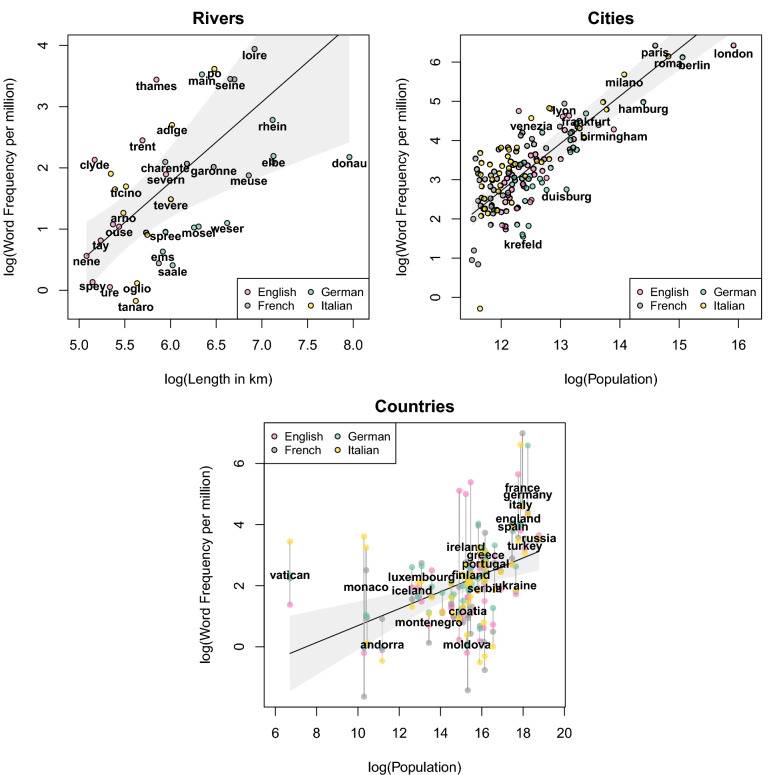
Relation between physical magnitude (river length, city and country population size, which would account for the relevance of these geographical entities) and logarithmic word frequency per million. The solid lines indicates the model prediction, the grey bands around it the 0.95-confidence interval.

Data were analyzed analogously to a behavioral experiment: The four languages (or the respective speaker populations) were considered as “participants” who produced a language corpus as a behavioural response. Thus, to analyze these data, we estimated Linear Mixed Effect Models (LMEMs) using the *lme4* package for R^25^. The models included a fixed effect for the log-transformed physical magnitude predictor (population size or river length), a random intercept for languages, as well as by-language random slopes for the predictor^26^. In the country study, due to the repeated-measures design, we additionally included a random intercept for the countries. We found that the physical magnitude significantly predicted the corresponding word frequency data across all studies (rivers: $b = 1.31$, $t(2.73) = 3.47$, $CI_{0.95} = [0.57, 2.05]$, $p = 0.047$; cities: $b = 1.21$, $CI_{0.95} = [1.06, 1.36]$, $t(17.55) = 15.71$, $p < 0.001$; countries: $b = 0.28$, $CI_{0.95} = [0.13, 0.42]$, $t(19.66) = 3.83$, $p = 0.001$; see Fig. 1).

In all models including only a single fixed effect parameter reported in this paper, a model comparison against an intercept-only model is significant exactly when the fixed effect parameter in the resulting model is significant. For all models reported in this paper, visual inspection of the model residuals revealed good fit of the models (see the Supplementary Material for more details).

### Discussion

In line with earlier findings^10^, Study 1 shows that there are multiple different cases in which we can find correlations between physical magnitude and word frequency. However, as in earlier studies, we cannot clearly distinguish between the physical size of the investigated entities (i.e., properties of the physical outside world) and their representational size (i.e., their relevance). Typically, more populous cities and countries as well as longer rivers are also more relevant to speaker communities. In this analysis, the Vatican is a noteworthy outlier (see Fig. 1), most likely due to its disproportionally high political, social, historical, cultural and especially religious influence as the seat of the papacy. This already suggests a dissociation between physical size and word frequencies; however, all these other factors for relevance/representational size are typically very difficult to measure in an objective way.

We now therefore turn to the human body, which (as discussed earlier) presents an ideal case for pitching the two against each other, with these two variables are completely independent from one another. For such an analysis, the results of Study 1 indicate that physical size can in principle predict word frequencies, and is thus a relevant factor we need to consider and control for in such an analysis, even if we suspect that representational size is the actual driving factor. Crucially, we measure both the physical size (as body surface area) and the representational size (as the “homunculus size”, i.e., the number of cortical stimulation points eliciting a response) in an objective and independent manner. We therefore don’t have to rely on behavioral measures such as relevance ratings (for which we wouldn’t know the factors influencing them) in order to predict the language behavior manifest in word frequencies. Importantly for this purpose, previous research has shown that the somatosensory homunculus provides a mental map of the body’s representation that influences judgments about our own and other’s bodies^27,28^, and can thus be used to approximate the mental representational size of the different body parts.

## Study 2: Word frequencies for human body parts

### Study 2a: Physical size of body parts

#### Methods

##### Body surface areas

Physical sizes for 25 different body parts (see Table 2) included in the study by Penfield and Boldrey^16^ were estimated as their proportional surface areas of a standardized human body with values expressing the percentage of body surface area, obtained from the seminal Lund and Browder chart^29^. This chart was originally created as a reference chart for burn areas but found widespread use in the literature on the determination of body surfaces (e.g.^30–32^). Areas for body parts not included in this chart were obtained graphically using the SAGE II Burn Diagram software (https://www.sagediagram.com), an online tool to quantify burn surface area that is based on the Lund and Browder chart attributes^33^. The SAGE Diagram is becoming increasingly used in the literature on burn injuries as it is particularly useful when computing the burn surface area when more than one body part is involved. Values for lateralized body parts (such as *thumb* or *hand*) were always extracted from the right body-side of the anterior view, and left body-side of the posterior view. Surface area estimates for body parts not available in neither of these sources were estimated from the literature (*teeth*^34^; *tongue*^35^, each averaged over males and females), and scaled to the surface area of the whole body (^36^; estimated using Mosteller’s formula^37^, with a normal-weight sample).

**Table 2:** The actual physical size, sensory and motor representational sizes, and average logarithmic word frequencies per million across all 30 languages for the 25 body parts investigated in this study (for detailed descriptions, see the Methods sections for Study 2 and 3).

Body part	Actual size	Sensory representation	Motor representation	Word frequency
	(% Body surface)	# Cortical stimulation points		log(WF per million)
Ankle	1.04	–	0.93	1.336
Arm	4.00	5.32	–	4.567
Back	13.00	–	0.47	4.090
Brow	0.08	–	0.80	1.829
Elbow	1.26	0.70	5.25	1.558
Eye	0.20	0.68	2.71	5.402
Face	2.14	4.12	–	5.152
Finger	0.86	7.65	3.77	3.822
Foot	3.50	0.92	–	4.897
Forearm	3.00	1.90	–	0.976
Hand	2.50	10.68	10.67	6.032
Hip	4.30	1.04	0.97	1.631
Jaw	1.12	–	3.45	1.480
Jaw and teeth	1.37	4.39	–	4.144
Knee	2.32	–	1.16	2.985
Leg	16.50	2.57	–	4.297
Lip	0.07	5.90	7.92	3.065
Nose	0.13	0.90	–	3.558
Shoulder	1.94	1.49	1.96	3.288
Throat	1.00	1.40	–	2.613
Thumb	0.30	3.79	2.34	1.687
Toe	0.62	0.64	0.94	2.268
Tongue	0.16	9.52	1.62	4.154
Trunk	13.00	2.04	–	2.058
Wrist	0.40	0.75	3.30	1.641

To ensure the robustness of our study and results, we applied different possible conceptualizations of body surface area: We calculated areas for all pairwise combinations of orientation (anterior surface only, except for *elbow*, where the posterior value was taken, versus anterior plus posterior surface) and lateralization (only the right body-side surface for paired body parts such as *hand*, versus the combined surface size for paired body parts). Additionally, two possible definitions of *arm* were considered: The standard anatomical definition applied by Penfield and Boldrey^16^ (the part between the shoulder and the elbow), and the common usage of the word (the part between shoulder and wrist).

##### Word frequencies

Only single-word names were included in our study, in order to obtain reliable word frequency estimates. For this purpose, we collapsed the values for the four fingers other than the thumb (*little finger*, *ring finger*, *middle finger*, and *index finger*) into a single item (*finger*). The English word labels for these body parts were translated into French, Italian, and German by native speakers. Not all words have single-word translations in all languages; these cases were handled as missing values. We chose not to consider the word contexts (for example, whether the words were used in a literal or figurative manner, as in *on the other hand*), to not introduce degrees of freedom in our analysis that ultimately depend on researcher intuition.

As in Study 1, word frequencies for English, German, French, and Italian were extracted from the large-scale WaCKy web corpora^23^. We considered word frequencies at the lemma level, that is, independent of morphological inflections. To capture possible parsing errors, we also extracted word frequencies for the words’ plural forms, where applicable. The frequency for the item *jaw and teeth*^16^ was estimated as the sum of the frequencies for *jaw* and *teeth*.

#### Results and discussion

We analyzed the data using an analogous model to the ones reported in Study 1: Word frequencies were predicted with a LMEM including random intercepts for body parts, random intercepts for the four languages, as well as by-language random slopes for the physical surface size^26^. Here, we are reporting the analysis for the anterior orientation, considering the combined area of paired body parts, and the anatomical shoulder-to-elbow definition of arm.

In this model, we found no significant relationship between the physical size of the body parts and their word frequency ($b = 0.05$, $CI_{0.95} = [-\, 0.08, 0.19]$, $t(25.80) = 0.81$, $p = 0.426$). This pattern of results reported remains the same for all eight possible conceptualizations of actual body size (*p*s between 0.355 and 0.718; this pattern also remains unchanged if we additionally considered the words *breast* and *stomach* which are not included in the study by Penfield and Boldrey^16^).

These results demonstrate that, unlike for the examples in Study 1, information about the physical size of human body parts is *not* encoded in word frequencies. Thus, not all types of information about the physical world can simply be decoded from language statistics, which importantly restricts the domain of previous results and conclusions^10,11^. However, this does not imply that our use of body part words follows an arbitrary distribution. In the second part of this study, we will demonstrate that the distortions observed so far are indeed systematic, and retrace the iconic cortical homunculus^16^, indicating therefore that word frequency encodes the functional relevance of body parts.

### Study 2b: Functional relevance and word frequencies of body parts

#### Methods

This study employs the same measures for word frequencies and actual physical sizes of the body parts as described in Study 2a. In addition, we obtained the following measures for representational body part sizes.

##### Representational body part size: number of cortical stimulations eliciting a response

The sensory representational sizes for the body parts, measured as the proportional number of cortical stimulations points (anterior as well as posterior to the fissure of Rolando) eliciting tactile sensation in the corresponding body parts, were extracted from the seminal study by Penfield and Boldrey^16^. While there surely are limitations to the original homunculus measures, current alternatives such as transcranial magnetic stimulation or high field functional magnetic resonance imaging come with their own limitations and do not necessarily provide better measures^17^. In addition to this, we focused on the measures by Penfield and Boldrey^16^ as it is the one simultaneously targeting the largest number of body parts in humans. Because there are some inconsistencies between the text and the figures of this article^17^, the values were extracted from Fig. 27 of the article, using graphical methods (overlaying them with colored bars and automatically determining their lengths). Because *taste* is not a body part, this item was omitted from our dataset.

Using the same graphical method, we also extracted the motor representational sizes for the body parts from Fig. 26 of ^16^, in this case measured as the proportional number of cortical stimulations points eliciting motor responses in the corresponding body parts. Since *swallow* and *vocalization* are not body parts, these items were omitted from our dataset.

#### Results and discussion

We found no significant relationship between the sensory representational body part sizes and the real body part surface areas, irrespective of all the possible definitions of the latter (i.e., anterior surface vs. anterior and posterior surface; midsagittal half vs. whole body) ($r = -\, 0.08 \text { to } -\, 0.12$, $p = 0.621 \text { to } 0.729$), highlighting the clear contrast between the two. The same was the case for motor representational body part sizes ($r = -\, 0.15 \text { to } -\, 0.23$, $p = 0.392 \text { to } 0.592$).

The LMEMs to analyze this data included random intercepts for body parts, random intercepts for the four languages, as well as by-language random slopes for the representational size and actual surface size^26^. Here, we again report the analysis for the anterior orientation, considering the combined area of paired body parts, and the anatomical shoulder-to-elbow definition of arm; however, the pattern of results reported remains highly similar for all eight possible conceptualizations of actual body size.

When analyzing data for the 20 items for which sensory representational sizes are available, a fixed effect for actual surface size did again not predict the word frequency data ($b = -\, 0.002$, $CI_{0.95} = [-\, 0.15, 0.14]$, $t(19.97) = -\, 0.03$, $p = 0.974$), as reported for the complete item set of 25 words in Study 2a. This parameter was included here as a baseline parameter, to ensure that any effects of representational size cannot be attributed to a residual correlation with physical body size. Additionally including such a fixed effect parameter for sensory representational size indeed significantly improved this baseline model, ($\chi ^2(1) = 5.40$, $p = 0.020$; with $b = 0.23$, $CI_{0.95} = [0.05, 0.41]$, $t(20.01) = 2.49$, $p = 0.022$ for its parameter in the resulting model). As a control analysis, we ran the same analysis without the models including any parameters for physical body size; the results stayed the same ($\chi ^2(1) = 5.33$, $p = 0.021$; with $b = 0.22$, $CI_{0.95} = [0.05, 0.40]$, $t(20.01) = 2.47$, $p = 0.023$ for the sensory representational size parameter). These results indicate that the statistical structure of language resembles the functional relevance of body parts.

For the 16 items for which motor representational sizes are available, a fixed effect for actual surface size does also not predict word frequencies ($b = 0.14$, $CI_{0.95} = [-\, 0.12, 0.39]$ , $t(17.15) = 1.06$, $p = 0.306$). In this case, however, additionally including a fixed effect for motor representational size did not significantly improve this model, although the *p*-value approached the borderline of the significance level ($\chi ^2(1) = 3.64$, $p = 0.057$; with $b = 0.23$, $CI_{0.95} = [0.01, 0.46]$, $t(15.88) = 2.02$, $p = 0.060$, for its parameter in the resulting model). In the control analysis without any parameters for physical body size, however, the results more clearly indicate non-significance of the motor representational size parameter ($\chi ^2(1) = 2.25$, $p = 0.134$; with $b = 0.19$, $CI_{0.95} = [-\, 0.05, 0.43]$, $t(16.00) = 1.55$, $p = 0.140$ for the parameter).

When analyzing the 11 items for which both sensory and motor representational sizes are available, a fixed effect for actual surface size does again not predict word frequencies ($b = -\, 0.07$, $CI_{0.95} = [-\, 0.78, 0.60]$, $t(11.54) = -\, 0.245$, $p = 0.811$). Additionally including a parameter for sensory representational size improved this model ($\chi ^2(1) = 4.61$, $p = 0.032$; and $\chi ^2(1) = 4.87$, $p = 0.027$ in a model without an actual surface size parameter); on the other hand, additionally including a parameter for motor representational size did not improve this model ($\chi ^2(1) = 1.99$, $p = 0.158$; and $\chi ^2(1) = 2.04$, $p = 0.153$ in a model without an actual surface size parameter). Including both representational size parameters did not improve any of the previously described models. Thus, when both representational sizes are considered, only the sensory parameter is predictive.

## Study 3: A large cross-linguistic validation

**Figure 2:**
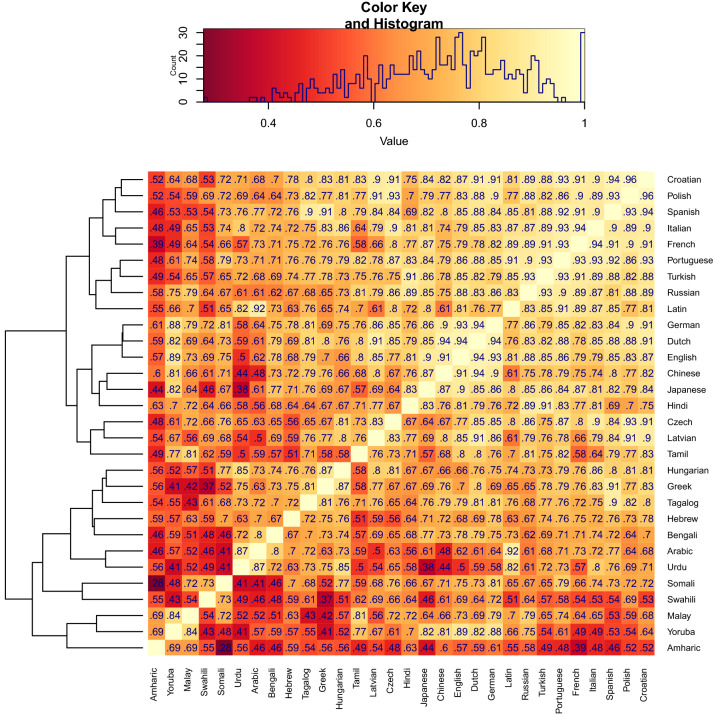
Between-language correlations of the logarithmic word frequencies per million, for the 25 words referring to body parts in Penfield and Boldrey^16^. The dendogram on the left side groups languages by their correlational patterns with other languages.

Up to this point, the languages we considered are very similar, both linguistically (two Germanic and two Romance languages) and culturally (Central Western Europe). To increase the generalizability and robustness of our results, we thus extended our analysis to a large number of languages from very different families and sub-families from all around the world, reaching a total of 30 languages. Since these languages include the world’s most-spoken native languages (such as Chinese, Hindi, Arabic, English, and Spanish), their combined native speaker populations cover more than 4 billion speakers (i.e., more than half of the world population). Correlations between the frequencies of words referring to body parts are generally very high across all languages, with a few pair-wise exceptions (see Fig. 2). This already suggests that common language- and culture-invariant factors affect the frequencies of words referring to body parts (with representational size being one such candidate). Besides the commonalities however, there are still structural differences and certain language clusters, which can be explored in more detail in future dedicated studies.

### Methods

In addition to the four languages described in Study 1 and 2, in Study 3 we considered word frequencies from 26 languages from all around the world and from different families and sub-families. These were all the languages for which (a) structurally comparable WaCKy or TenTen corpora were available (see the next paragraph), an (b) for which we were able to find a reliable native-speaking informant. An overview of these languages is provided in Table 1. All body part labels were translated by native speakers, except for Latin. All items were back-translated and checked by the authors using online dictionaries.

The systematically evaluated WaCKy corpora^23^ are not available in these languages; however, Kilgarriff et al.^38^ present a framework that produces web corpora using an extremely similar algorithm, thus extending the core set of WaCKy corpora (providing us with Spanish, Croatian, Latvian, Greek, Turkish, Urdu, Hindi, Bengali, Hebrew, Amharic, Somali, Swahili, Yoruba, Tamil, Malaysian, and Japanese corpora). In cases where these were not available (Dutch, Portuguese, Russian, Polish, Czech, Hungarian, Arabic, Tagalog, and Chinese), we instead employed the corpora from the TenTen family, collected using an extension of the WaCKy algorithm^39^. Thus, all modern-language corpora were collected from structurally similar sources (websites), using very similar algorithmic frameworks. For Latin, we employed the historical LatinISE corpus^40^. Word frequencies for these 26 additional languages were collected using the Sketch Engine tool^41^. Not all words have single-word translations in all languages, and in some cases translations were not found in the respective language corpora; in order to obtain reliable and comparable frequency estimates for all words, these cases needed to be handled as missing values. Again, we computed (for all 30 languages) word frequencies per million words, which were then logarithmized^24^. To ensure that these values are similarly reliable for these corpora of very different size (see Table 1), we derived them for the first 3 million words (the size of the smallest corpus, YorubaWaC) in the four WaCKy corpora used in Study 1 and 2, and compared them to the values derived from the whole corpora. We consistently observed correlations of $r > 0.967$.

### Results

Some languages use the same word for different body parts (e.g. *ruka* for hand and arm in Croatian). The results reported in this section remain unchanged if such cases are excluded from the analysis.

**Figure 3:**
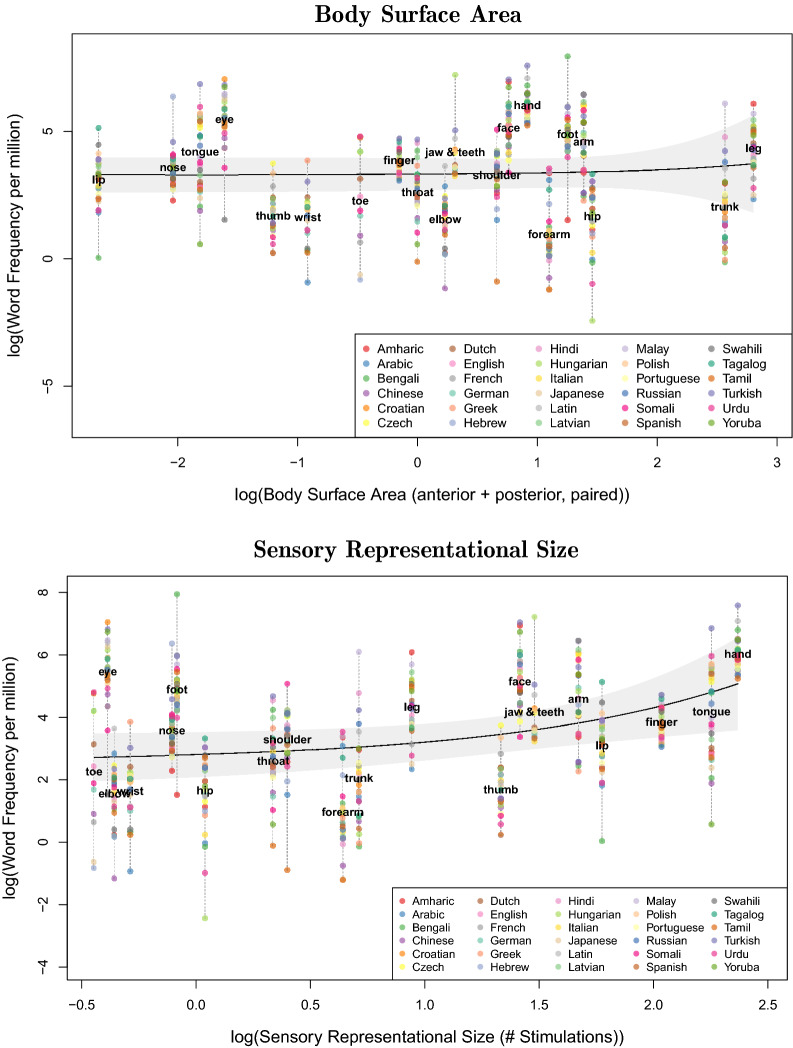
Relation between logarithmic word frequency per million and body surface area (*upper panel*) or sensory representational size (*lower panel*), as measured by the number of stimulation points eliciting a tactile senstation (Penfield and Boldrey^16^). For visual clarity, the x-axes are displayed on a logarithmic scale. The solid lines indicate model predictions, the gray bands around it 0.95-confidence intervals.

For the 20 items for which sensory data is available, a fixed effect for actual surface size did not predict the word frequency data across all 30 languages ($b = -\, 0.01$, $CI_{0.95} = [-\,0.16, 0.14]$, $t(20.73) = -\,0.13$, $p = 0.898$; see Fig. 3) in a LMEM including random intercepts for the languages, by-language random slopes for the representational size and actual surface size, and random intercepts for the body parts. Yet, additionally including a fixed effect for sensory representational size again significantly improved the model ($\chi ^2(1) = 5.34$, $p = 0.021$; with $b = 0.23$, $CI_{0.95} = [0.05, 0.42]$, $t(20.00) = 2.47$, $p = 0.023$ for its parameter in the resulting model; see Fig. 3). This pattern is invariant against the concrete operationalization of physical body part size. It also remains the same for a model that does not contain parameters for actual surface size ($\chi ^2(1) = 5.20$, $p = 0.023$; with $b = 0.23$, $CI_{0.95} = [0.04, 0.41]$, $t(20.00) = 2.44$, $p = 0.024$ for the sensory representational size parameter).

For the 16 items for which motor data is available, a fixed effect for actual surface size again does not predict word frequencies ($b = 0.09$, $CI_{0.95} = [-\,0.13, 0.32]$, $t(16.46) = 0.82$, $p = 0.427$). Additionally including a fixed effect for motor representational size significantly improved this model ($\chi ^2(1) = 3.98$, $p = 0.046$; with $b = 0.25$, $CI_{0.95} = [0.02, 0.47]$, $t(16.01) = 2.12$, $p = 0.0496$, for its parameter in the resulting model). This analysis is mostly invariant against the different conceptualizations of body surface area, except for the anterior plus posterior orientation considering the combined area of paired body parts: With this operationalization, the effect of motor representational size fails to reach significance ($p = 0.056$). In addition, the motor representational size parameter is not significant when the model does not already contain parameters for actual surface size ($\chi ^2(1) = 2.89$, $p = 0.089$; with $b = 0.21$, $CI_{0.95} = [-\,0.02, 0.45]$, $t(16.00) = 1.78$, $p = 0.0946$, for its parameter in the resulting model. Hence, in line with Study 2, there is no solid empirical evidence suggesting an effect of motor representational size.

For the 11 items for which both sensory and motor representational sizes are available, a fixed effect for actual surface size does again not predict word frequencies ($b = -\,0.08$, $CI_{0.95} = [-\,0.79, 0.63]$, $t(11.01) = -\,0.225$, $p = 0.826$). We again observe the same pattern as in Study 2: Including a parameter for sensory representational size improved this model ($\chi ^2(1) = 4.81$, $p = 0.028$; and $\chi ^2(1) = 4.85$, $p = 0.028$ in a model without an actual surface size parameter), while additionally including a parameter for motor representational size did not improve this model ($\chi ^2(1) = 2.45$, $p = 0.117$; and $\chi ^2(1) = 2.42$, $p = 0.120$ in a model without an actual surface size parameter). Again, including both parameters did not improve any of the previously described models.

**Figure 4:**
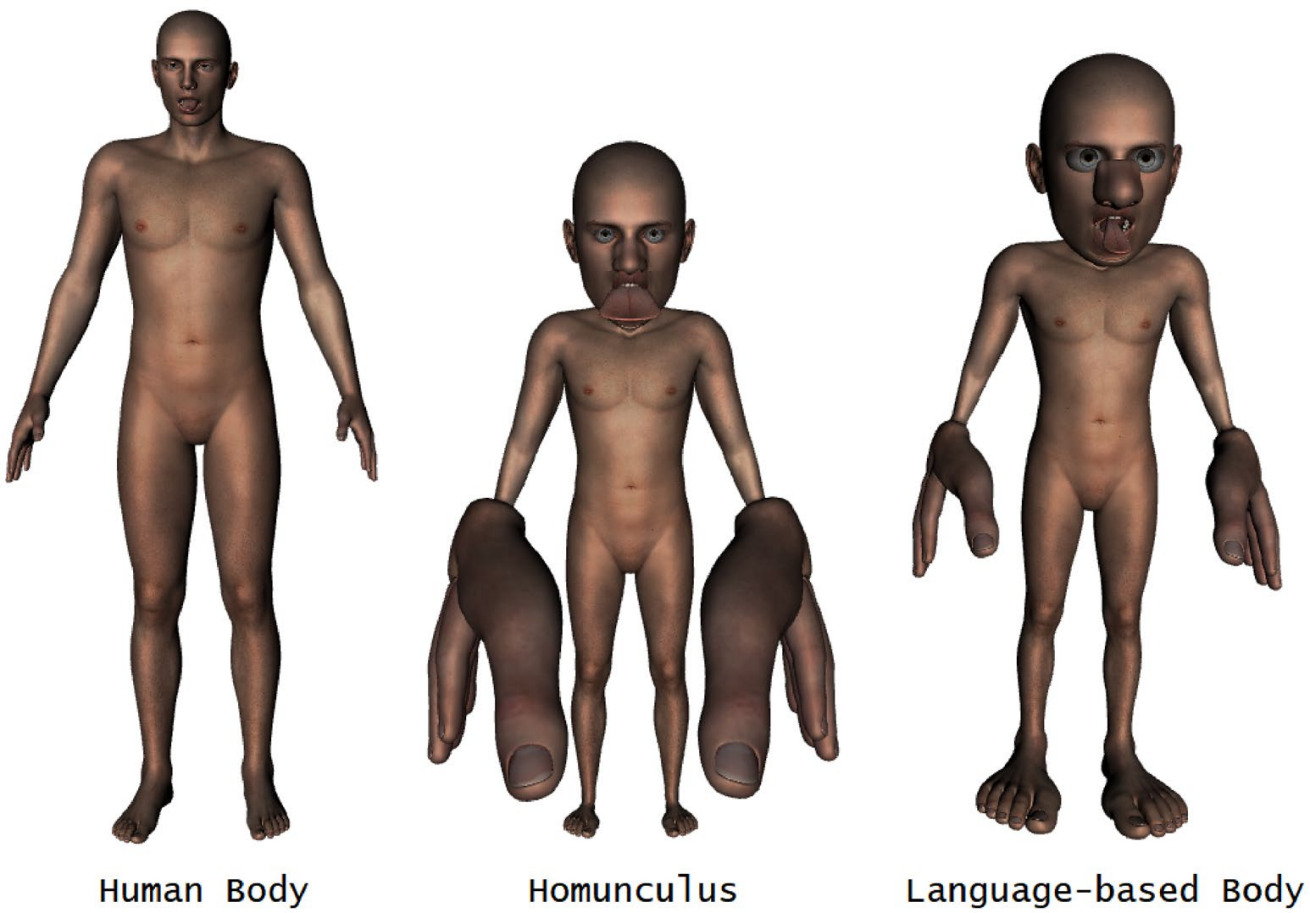
*Left* Actual human body proportions. *Middle* Sensory representational size proportions (i.e., the sensory homunculus (1)). *Right* Word frequency proportions. These figures were created by computing the relative (distorted) surface area of each body part for stimulations (Homunculus) and word frequencies (Language-based body), and mapping them on the “Genesis 2 Male” model in Daz 3D (https://www.daz3d.com/). Yet, because the morphing software used is based on volume rather than on surface area, we further adjusted our computation to the different ratios of surface area to the volume of distinct body parts^42^.

For a graphical display of the actual body proportions, the proportions according to sensory representational sizes (i.e., the somatosensory homunculus), and the word frequency proportions (aggregated across all 30 languages), as displayed in Table 2, see Fig. 4. As can be seen in this figure, the language-based body retains many of the distortions of the sensory homunculus, thus suggesting that linguistic frequency encodes the functional relevance of body parts rather than their size.

## General Discussion

The first study presented here initially seems to corroborate the view that physical properties of the outside world can be retrieved from statistical patterns of language use, namely word frequencies, replicating earlier results on city sizes^10^ and extending them to other domains (country sizes and river lengths). However, employing the human body as an ideal test case, the second study clearly demonstrates the limitations of this relation: Here, word frequencies are heavily distorted with respect to actual physical properties. Critically, they instead systematically align with the way in which our cortical and cognitive representations of our own body, as illustrated by the somatosensory homunculus^16^, are themselves heavy distortions of its physical dimensions.

Thus, we identified a counter-example demonstrating that a general connection between language statistics and the physical outside world does not exist. Cases in which such information can be decoded from language^11^ rely on the fact that properties of the physical world are often to some degree retained in our representations of it, and thus indirectly transmitted to language. However, as shown in the present study, not even this indirect connection is reliable: Generally speaking, one cannot re-construct the world humans live in from statistical analyses of their vast collections of text—only the way in which it is filtered, distorted and biased through the minds of speakers. However, the present results—that human representations of the world rather than physical realities are reflected in surface-level language statistics—are not necessarily restricted to body parts. This is already exemplified in our first study by the frequency of the Vatican city: the high political, social, historical, cultural and especially religious influence of the Vatican would likely determine its high frequency in language, despite its very small population size.

Note that, despite one may wonder whether representational size is just another type of physical size, this variable actually measures the number of cortical stimulation points eliciting a (sensory) response. Thus, the variable is defined in functional terms, as a relation between brain tissue and behavior—and just by looking at the brain tissue alone, it would not be possible to determine the representational size in the somatosensory homunculus. Even if this was possible by inspecting all neural pathways between the respective body parts and the cortex, one would still need to assume that there is a fundamental difference at all between mental representations on the one hand and the brain on the other hand—an intensely debated question in the controversy over the mind-body problem^43^.

In the present study, we examined both sensory and motor representational sizes as measured by Penfield and Boldrey^16^. Our findings indicate a clear pattern for sensory representational sizes, which consistently predict word frequencies on their own, and emerge as the only predictive variable when analysed simultaneously with motor representational sizes. On the contrary, the results for motor representational sizes do not clearly indicate an effect of this variable. While it should be noted that the item set for motor representational sizes is smaller, and especially the direct comparisons between sensory and motor sizes run on a fairly restricted set of 11 items, motor representational size cannot be interpreted to reliably predict word frequencies. In this context, we want to note that a direct comparison between sensory and motor representational size was not the aim of the present study, which focused on the divergence between physical size and (functional) relevance.

Of course, sensory and motor representational size is not the only factor establishing the relevance of a body part, and not the only predictor of word frequency. For example, the *eyes* in Study 2 and 3 have the second-highest word frequency (after *hand*), even though they are very small both physically as well as in terms of sensory representational size. However, eyes are very relevant to humans both as the organ responsible for our most important perceptual sense—vision—and for social interaction (a similar argument can be made for *face*). Another case can be made for the high frequency associated with *foot*. Feet can be in fact literally considered as the foundation of the human body: they allow balance and posture, and constitute the lowest extremity of the body’s vertical axis of reference imposed by gravity (e.g., head–foot). This is also reflected in language, as many metaphorical projections are not arbitrary but rather constrained by our prototypical bodily posture (e.g., *land on one’s feet* means to be in good condition after having a difficult experience^44^). Perhaps more importantly, beyond their central role in postural balance, feet also enable us to navigate the surrounding environment. As another example for potentially relevant factors beyond sensory relevance, the human body has a strong power to orient and attract visual attention. Faces and body parts are stimuli of great biological and social significance: eye movements in natural viewing conditions are often directed to human faces, with the eye region being particularly crucial for face recognition^45^. Not only the face, but also other body parts attract the viewer’s attention, especially when the exploration pattern involves the simulations of actions^46^. As such, the distribution of attentional resources over different body parts in natural scene perception may be another factor contributing to word relevance and consequently frequency. Hence, sensory relevance may be just one among other factors contributing to word relevance, and there is much room for future research on other possible factors.

Notably, terms referring to body parts—which provide a highly salient piece of common ground for all speakers^47^—are especially prone to various types of polysemy, such as metaphorical or metonymic extension (for example, in expressions such as *on the other hand*, *head of state*, or *a shoulder to cry on*^48^). The human body is indeed a very important source for linguistic expressions such as metaphors^49^: For instance, across many cultures and languages human emotions are normally referred to by metaphors derived from names for various body parts. Similarly, metaphors in English or German connect human body parts and their physiological functions with the sphere of politics^50^. This affinity for polysemy is a plausible factor contributing to the observed relation between representational size and word frequency, when terms for particularly salient body parts (such as *hand* or *face*) are also more frequently used in such non-literal meanings. To our knowledge, we currently do not have high-performing fully-automated tools to reliably identify literal versus non-literal word uses in all language corpora, which is why we considered all instances of a word for computing word frequencies. Empirically investigating to what extent the relation between relational size and word frequency is driven by literal versus non-literal word uses thus remains an interesting question for future research.

Our results are also relevant in light of the distinction between “primary” sensorimotor representations of one’s own body on the one hand, and language as a secondary representation system^51,52^. While these have traditionally been studied independently from one another, researchers from different fields have started to close this gap by showing links between the two systems^47^. As stated by Dingemanse^47^,

What is interesting about this specific case however is that something that is in essence private (namely, sensory-motor representation) can apparently be of joint salience to speaker and hearer. This really reveals the power of the secondary representation system, in that it affords its users the possibility to tap into the resources of personal experience. Body-part terms thus are a special kind of linguistic sign: they represent the intertwining of the private system of sensory-motor representation on the one hand, and the public, socially constituted system of human language on the other hand. (p. 2133)

With our large-scale quantitative approach, we are able to provide clear quantitative empirical evidence about the extent to which these systems are intertwined.

To conclude this article, we hark back to our initial thought experiment: what would the group of alien scientists be able to learn solely from the statistical structure of language? Which type of information is encoded in this surface-level language data? We argued that, as the direct product of the brains and minds of speakers, the statistical structure of language is necessarily subject to their biases and representational distortions. Thus, the alien scientists would learn about the human representation of the world rather than about the world itself (of course, whether or not the alien scientists would be *aware* of this depends on them having at least some independent mean of verification, such as presented in this study). In this respect, our findings are in line with others^53,54^, showing how patterns of language use capture mental representations and cognitive biases in other domains, such as stereotypes towards social groups^53,54^. As a direct consequence however, this also means that an outside observer can learn about human representations of the world from a directly-observable human artefact (here, corpora of written text), without the need to actually encounter humans, talk to them, or perform psychological studies with them. Thus, patterns of language use open a window into the brain and mind of language users, with recorded or written language providing this opportunity even without requiring the synchronical presence of actual speakers. Therefore, in a period of time where producing and making publicly available large amounts of text via digital platforms is becoming standard practice for a large portion of the human population, we speakers need to be aware that we are more and more pushing this window open.

## Supplementary Information

Supplementary Information.

## References

[1] Bender, E. M. & Koller, A. Climbing towards NLU: On meaning, form, and understanding in the age of data. In *Proc. 58th Annual Meeting of the Association for Computational Linguistics*, 5185–5198 (2020).

[2] Louwerse MM (2011). Symbol interdependency in symbolic and embodied cognition. Top. Cogn. Sci..

[3] Günther F, Rinaldi L, Marelli M (2019). Vector-space models of semantic representation from a cognitive perspective: A discussion of common misconceptions. Perspect. Psychol. Sci..

[4] Rinaldi L, Marelli M (2020). Maps and space are entangled with language experience. Trend Cogn. Sci..

[5] Searle JR (1980). Minds, brains, and programs. Behav. Brain Sci..

[6] De Vega M, Glenberg A, Graesser A (2012). Symbols and Embodiment: Debates on Meaning and Cognition.

[7] Vega MD, Glenberg AM, Graesser AC (2008). Symbols and Embodiment: Debates on Meaning and Cognition.

[8] Cangelosi A, Riga T (2006). An embodied model for sensorimotor grounding and grounding transfer: Experiments with epigenetic robots. Cogn. Sci..

[9] Lakoff G, Johnson M (1980). Metaphors We Live By.

[10] Louwerse MM, Zwaan RA (2009). Language encodes geographical information. Cogn. Sci..

[11] Recchia GL, Louwerse MM (2016). Archaeology through computational linguistics: Inscription statistics predict excavation sites of Indus valley artifacts. Cogn. Sci..

[12] Louwerse MM (2008). Embodied relations are encoded in language. Psychon. Bull. Rev..

[13] Connolly AC, Gleitman LR, Thompson-Schill SL (2007). Effect of congenital blindness on the semantic representation of some everyday concepts. Proc. Natl. Acad. Sci..

[14] Lenci A, Baroni M, Cazzolli G, Marotta G (2013). BLIND: A set of semantic feature norms from the congenitally blind. Behav. Res. Methods.

[15] Johns BT, Jones MN (2012). Perceptual inference through global lexical similarity. Top. Cogn. Sci..

[16] Penfield W, Boldrey E (1937). Somatic motor and sensory representation in the cerebral cortex of man as studied by electrical stimulation. Brain.

[17] Catani M (2017). A little man of some importance. Brain.

[18] Piantadosi ST (2014). Zipf’s word frequency law in natural language: A critical review and future directions. Psychon. Bull. Rev..

[19] Zipf GK (1949). Human Behavior and the Principle of Least Effort.

[20] Brysbaert M, Mandera P, Keuleers E (2018). The word frequency effect in word processing: An updated review. Curr. Dir. Psychol. Sci..

[21] Bates E (2003). Timed picture naming in seven languages. Psychon. Bull. Rev..

[22] Central Intelligence Agency (2008). The World Factbook.

[23] Baroni M, Bernardini S, Ferraresi A, Zanchetta E (2009). The WaCky wide web: A collection of very large linguistically processed web-crawled corpora. Lang. Resour. Eval..

[24] Van Heuven WJ, Mandera P, Keuleers E, Brysbaert M (2014). SUBTLEX-UK: A new and improved word frequency database for British English. Q. J. Exp. Psychol..

[25] Bates D, Mächler M, Bolker B, Walker S (2015). Fitting linear mixed-effects models using lme4. J. Stat. Softw..

[26] Barr DJ, Levy R, Scheepers C, Tily HJ (2013). Random effects structure for confirmatory hypothesis testing: Keep it maximal. J. Mem. Lang..

[27] Linkenauger SA (2015). The perceptual homunculus: The perception of the relative proportions of the human body. J. Exp. Psychol. Gen..

[28] Longo MR, Azañón E, Haggard P (2010). More than skin deep: Body representation beyond primary somatosensory cortex. Neuropsychologia.

[29] Lund CC, Browder NC (1944). The estimation of areas of burns. Surg. Gynecol. Obstetr..

[30] Prieto MF, Acha B, Gómez-Cıa T, Fondón I, Serrano C (2011). A system for 3D representation of burns and calculation of burnt skin area. Burns.

[31] Wachtel TL, Berry CC, Wachtel EE, Frank HA (2000). The inter-rater reliability of estimating the size of burns from various burn area chart drawings. Burns.

[32] Yu C-Y, Lin C-H, Yang Y-H (2010). Human body surface area database and estimation formula. Burns.

[33] Richard R, Jones JA, Parshley P (2015). Hierarchical decomposition of burn body diagram based on cutaneous functional units and its utility. J. Burn Care Res..

[34] Collins L, Dawes C (1987). The surface area of the adult human mouth and thickness of the salivary film covering the teeth and oral mucosa. J. Dent. Res..

[35] Liégeois F, Albert A, Limme M (2009). Comparison between tongue volume from magnetic resonance images and tongue area from profile cephalograms. Eur. J. Orthod..

[36] Verbraecken J, Van de Heyning P, De Backer W, Van Gaal L (2006). Body surface area in normal-weight, overweight, and obese adults. A comparison study. Metabolism.

[37] Mosteller R (1987). Simplified calculation of body surface area. N. Engl. J. Med..

[38] Kilgarriff, A., Reddy, S., Pomikálek, J. & Avinesh, P. A Corpus factory for many languages. In *Proc. 7th Conference on International Language Resources and Evaluation (LREC’10)*, 904–910 (ELRA, 2010).

[39] Jakubıček, M., Kilgarriff, A., Kovář, V., Rychl, P. & Suchomel, V. The TenTen corpus family. In *Proc. 7th International Corpus Linguistics Conference*, 125–127 (2013).

[40] McGillivray B, Kilgarriff A (2013). Tools for Historical Corpus Research, and a Corpus of Latin in New Methods in Historical Corpora.

[41] Kilgarriff A (2014). The Sketch Engine: Ten years on. Lexicography.

[42] Tikuisis P, Meunier P, Jubenville C (2001). Human body surface area: Measurement and prediction using three dimensional body scans. Eur. J. Appl. Physiol..

[43] Robinson H, Zalta EN (2020). Dualism. The Stanford Encyclopedia of Philosophy.

[44] Škara D (2004). Body metaphors-reading the body in contemporary culture. Coll. Antropol..

[45] Royer J (2018). Greater reliance on the eye region predicts better face recognition ability. Cognition.

[46] Massaro D (2012). When art moves the eyes: A behavioral and eye-tracking study. PLoS ONE.

[47] Dingemanse M (2009). The selective advantage of body-part terms. J. Pragmat..

[48] Kraska-Szlenk I (2014). Semantic extensions of body part terms: Common patterns and their interpretation. Lang. Sci..

[49] Swan T (2009). Metaphors of body and mind in the history of English. Engl. Stud..

[50] Musolff A (2008). The embodiment of Europe: How do metaphors evolve. Body Lang. Mind.

[51] Tomasello M (1999). The Cultural Origins of Human Cognition.

[52] Keller R (1998). A Theory of Linguistic Signs.

[53] Bhatia S (2017). The semantic representation of prejudice and stereotypes. Cognition.

[54] Caliskan A, Bryson JJ, Narayanan A (2017). Semantics derived automatically from language corpora contain human-like biases. Science.

