# Supplementary material for: Language statistics as a window into mental representations
Source: Sci Rep. 2022 May 16;12:8043. doi: 10.1038/s41598-022-12027-5 (PMC9110419; doi:10.1038/s41598-022-12027-5)
Supplement: Supplementary file 1 — Supplementary Information. [file 41598_2022_12027_MOESM1_ESM.pdf]

Supplemenary Material: Additional details on the statistical models

Study 1

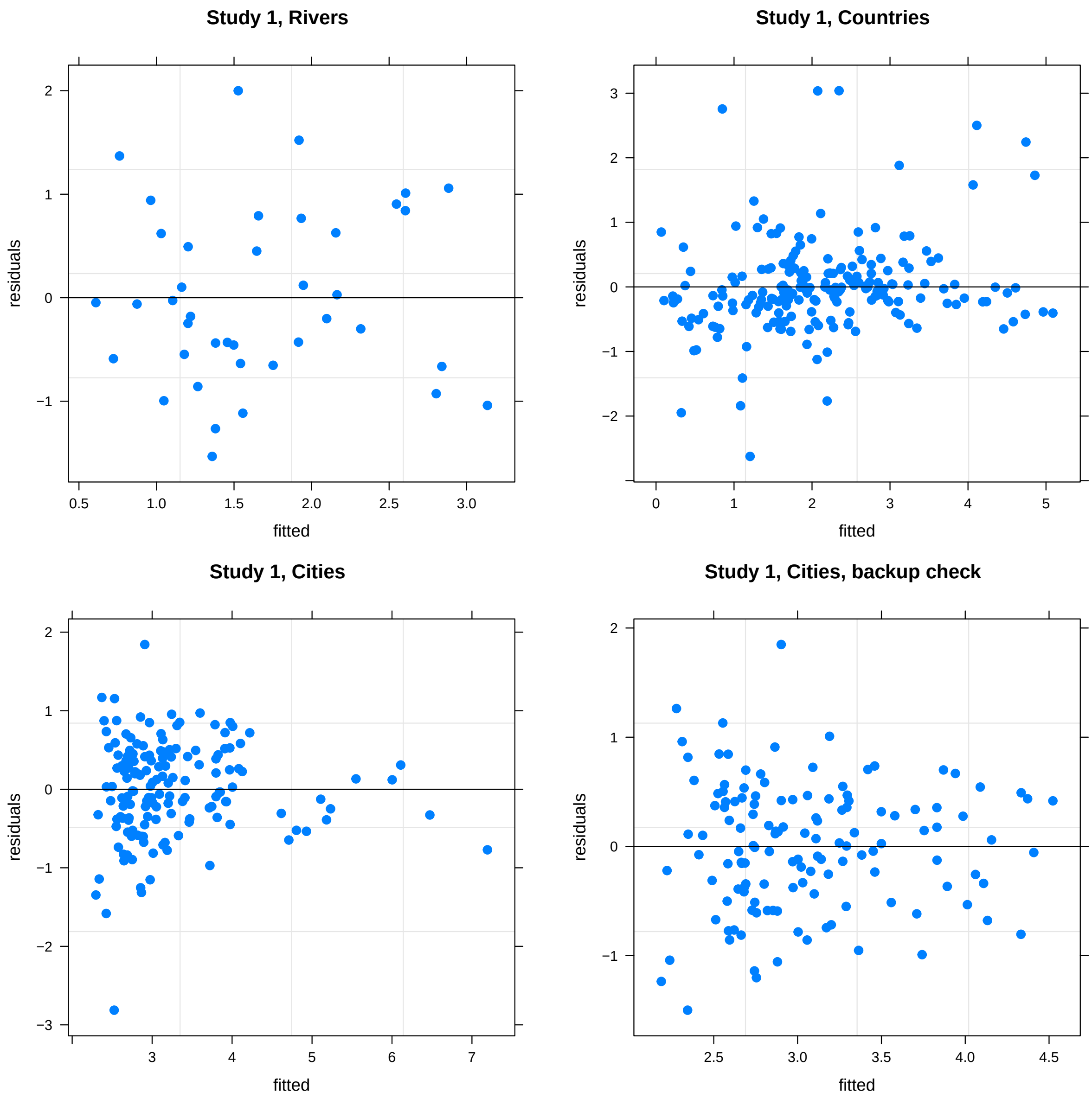

Figure S1. Residuals-versus-fitted plots for the LMEMs described in Study 1.

In Study 1, the residuals-versus-fitted plots look acceptable (i.e., residuals are relatively uniformly distributed across the range of fitted values) for most models (see Figure S1), for the most part indicating no clear violations of the assumptions of linearity or homoskedasticity. However, for the “cities” analysis”, the variance of residuals gets

smaller as the fitted values get larger (indicating a violation of homoskedasticity). To be sure that this did not affect the pattern of results, we ran the same analysis as described in the main text on a dataset from which we excluded the "problematic" items (i.e., those with fitted values > 4.5, and the item with a residual value of < -2). The results stay the same, with the parameter for physical magnitude (i.e., population size) at $t(2.65) = 9.93$, $p = .004$, but the residuals-vs-fitted plot looks far better in this case (see the "backup check" in Figure S1).

In the main text, we report parameters for the models already containing the parameters of interest the models with one fixed factor. For completion, we report model comparisons against intercept-only models here:

- rivers: $\chi^2(1) = 7.13$, $p = .008$, for the length parameter
- cities: $\chi^2(1) = 17.94$, $p < .001$, for the population parameter
- countries: $\chi^2(1) = 9.24$, $p = .002$, for the population parameter

Study 2

The residuals-versus-fitted plots for Study 2 are displayed in Figure S2, and indicate no clear violations of the assumption of linearity or homoskedasticity.

A model comparison between an intercept-only model and a model containing a fixed effect for actual surface size yields $\chi^2(1) = 0.580$, $p = .447$. All other model comparisons from there are reported in the main text.

Study 3

The residuals-versus-fitted plots for Study 3 are displayed in Figure S3, and again indicate no clear violations of the assumption of linearity or homoskedasticity.

A model comparison between an intercept-only model and a model containing a fixed effect for actual surface size yields $\chi^2(1) = 0.541$, $p = .462$. All other model comparisons from there are reported in the main text.

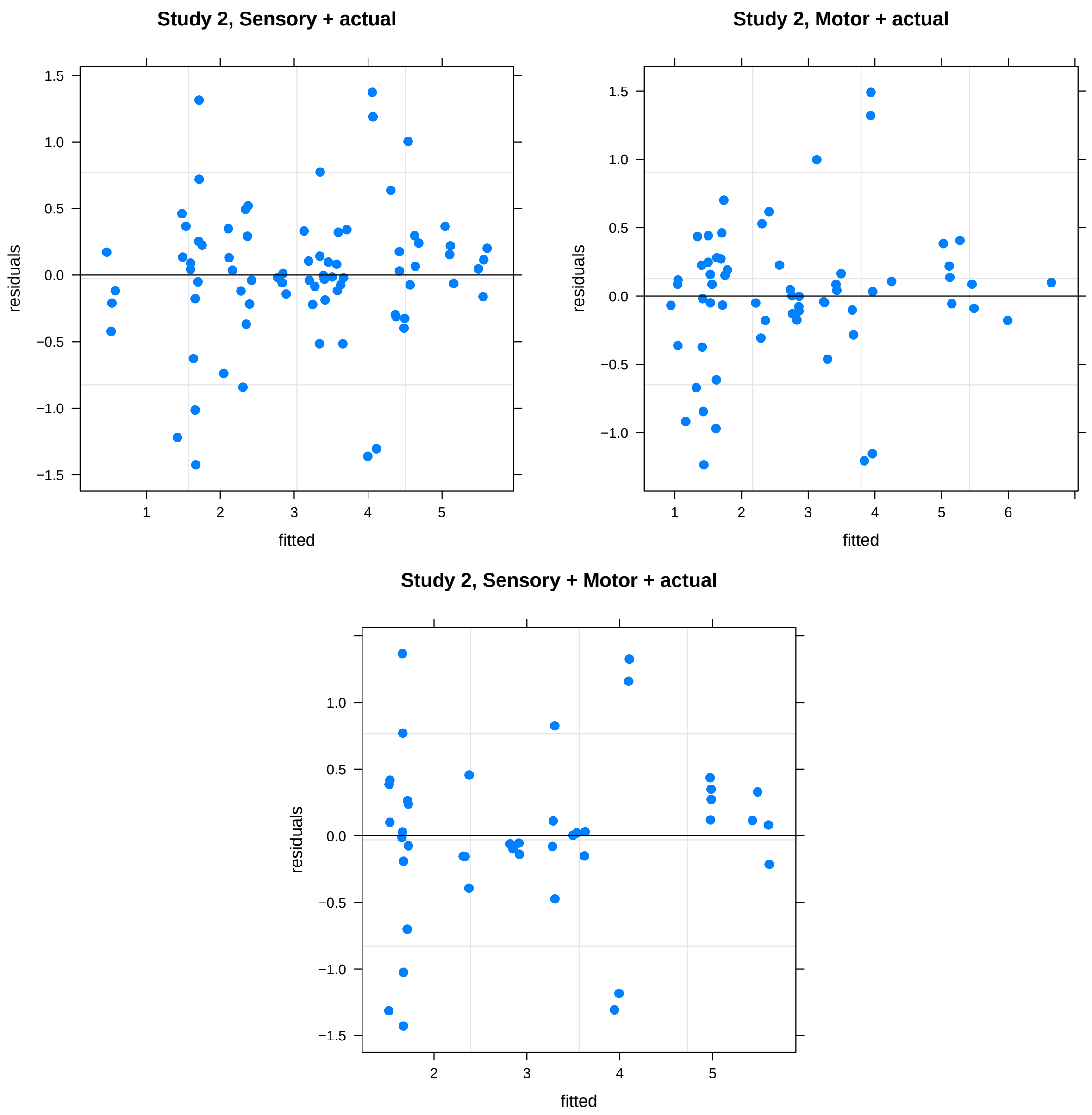

Figure S2. Residuals-versus-fitted plots for the LMEMs described in Study 2.

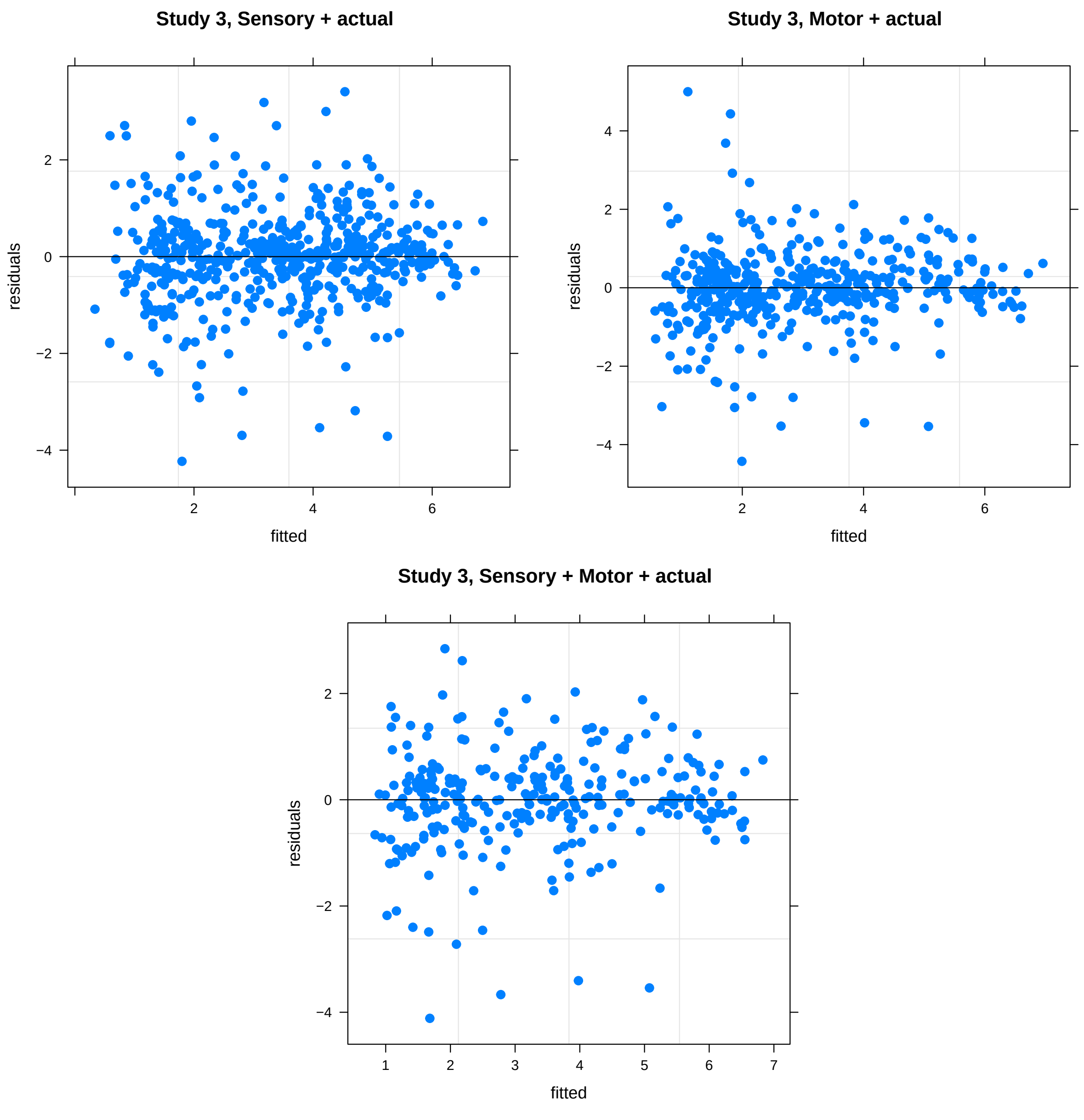

Figure S3. Residuals-versus-fitted plots for the LMEMs described in Study 3.
